# A novel approach to assess diet diversity: a development of the Nutritional Functional Diversity indicator

**DOI:** 10.3389/fnut.2023.1170831

**Published:** 2023-10-19

**Authors:** Matteo Di Maso, Francesca Bravi, Jerry Polesel, Luigino Dal Maso, Linia Patel, Carlo La Vecchia, Monica Ferraroni

**Affiliations:** ^1^Department of Clinical Sciences and Community Health, Branch of Medical Statistics, Biometry and Epidemiology “G.A. Maccacaro”, Università degli Studi di Milano, Milan, Italy; ^2^Unit of Cancer Epidemiology, Centro di Riferimento Oncologico di Aviano (CRO), IRCCS, Aviano, Italy; ^3^Fondazione IRCCS Ca’ Granda, Ospedale Maggiore Policlinico, Milan, Italy

**Keywords:** diet diversity, novel approach, cluster analysis, nutritional composition, diet quality, Mediterranean diet, Healthy Eating Index-2015

## Abstract

**Background and aims:**

Diversity is a key element of diet quality. The Food Variety Score (FVS) is used to assess diversity, especially in low- and middle-income countries. It sums up the number of foods consumed ignoring their nutrient content. A more suitable index should combine the number of foods consumed and their nutritional composition. We adapted the Nutritional Functional Diversity indicator (NFD), proposed by ecologists, to measure diversity in the human diet. We compared NFD and FVS evaluating subjects’ distributions across quartiles of the two diversity indices. To evaluate which one reflected a higher diet quality, we estimated associations between these two diversity indices and diet quality measures, i.e., the Mediterranean Diet Score (MDS) and the Healthy Eating Index-2015 (HEI-2015). Associations were expressed by odds ratios (OR) and corresponding 95% confidence intervals (CI).

**Materials and methods:**

We used the data of controls only derived from an integrated series of hospital-based case-control cancer studies conducted in different Italian areas. The NFD identifies groups of foods based on a set of nutrients according to a cluster analysis. Some steps are required: creating a food-nutrient matrix; clustering of the Euclidean food-food distance matrix to identify groups of foods with nutritional (dis)similarities; and calculating the NFD as the ratio between the sum of branch lengths of the dendrogram belonging to the number of foods consumed by individuals (i.e., subject-specific diversity) and the sum of all branch lengths of the dendrogram (i.e., maximal diversity).

**Results:**

More than one quarter of individuals (28.4%) were differently classified within quartiles of the two diversity indices. For both indices, increasing the diversity level increased the risk for adhering to MDS (OR for NFD = 11.26; 95% CI: 7.88–16.09, and OR for FVS = 6.80; 95% CI: 4.84–9.54) and to HEI-2015 (OR for NDF = 2.86; 95% CI: 2.39–3.42, and OR for FVS = 2.72; 95% CI: 2.27–3.26). Associations were stronger for NFD.

**Conclusion:**

Our findings showed a greater ability of NFD to assess diet quality quantifying the degree of diversity.

## 1. Introduction

For a long time, public health nutrition guidelines have recommended diversity (or variety) in eating patterns as a key element of an optimal diet (1, 2). The guidelines highlight that no single food or food group ensures an adequate intake of all necessary nutrients.

Several indices have been proposed to assess diversity (3–8). The most commonly used are the Food Variety Score (FVS) (3) and the Dietary Diversity Score (DDS) (4). These indices sum up the number of foods (FVS) or foods groups (DDS) consumed by an individual over a reference period, usually from 1 to 3 days (4, 9), but longer periods have also been considered (10, 11). Nevertheless, FVS would be equal for individuals who eat the same number of foods even if the foods consumed have an intrinsically different nutritional composition. Likewise, the DDS is not able to consider nutrient content of foods consumed within the same food group and between food groups. Due to their simplistic counting approach, FVS and DDS have been especially used in countries with limited food availability (e.g., low-income countries) (12).

Other composite measures of overall diet quality (13) have been suggested for assessing the dietary compliance to nutritional recommendations (14–17) or the adherence to *a priori* determined diets (18, 19). The use of diet quality measures became widespread in populations with more complex dietary patterns such as high-income countries (12). Although these measures provide an overall assessment of diet quality, they do not formally quantify the diversity of an individual’s diet.

A trade-off approach to assess diversity as a component of diet quality could consist of combining the information from the number of foods consumed and nutrient composition. Ecologists developed the Functional Diversity indicator to evaluate the impact of biodiversity among species (20). DeClerck and colleagues (21) extended Functional Diversity indicator to describe diversity of nutrients in cropping systems. Since adding of key species belonging to distinct nutritional groups on a farm or household system increases the availability of nutrients, DeClerk et al.’s Nutritional Functional Diversity indicator (NFD) links agrobiodiversity to diet according to the idea that cropping diversity can modify available nutrients for humans (21).

In this study, we adapted the NFD to assess diversity in the human diet. Furthermore, we compared NFD and FVS evaluating their differences in quantifying diet diversity. Finally, to evaluate which one reflected a higher diet quality, we estimated the associations between these two diversity indices and the Mediterranean Diet Score (MDS) and the Healthy Eating Index-2015 (HEI-2015), respectively.

## 2. Materials and methods

### 2.1. Subjects

We used the data of an integrated series of hospital-based case-control studies enrolling cancer patients and controls in different Italian areas from 1991 to 2008. These studies shared a similar protocol and a structured questionnaire to collect individual data. For the purposes of the present analysis, we considered only data of controls, for which individuals’ behaviors (including dietary ones) are more similar to the general population. Indeed, controls were hospital patients admitted for a wide spectrum of acute diseases or conditions not associated to long-term diet modifications. Trained interviewers administered a structured questionnaire to collect information on sociodemographic characteristics, anthropometric measures, lifestyle (e.g., smoking, physical activity), reproductive factors, use of drugs, personal history of disease, family history of cancer, and dietary habits. Habitual diet (i.e., 1 year prior to the interview) was assessed by a reproducible and validated food frequency questionnaire (FFQ) (22–25). The FFQ included information on weekly intake of foods (or recipes) and beverages according to the following sections: (1) milk, hot beverages and sweeteners; (2) bread, cereals and first courses; (3) second courses (e.g., meat and other main dishes); (4) side dishes (i.e., vegetables); (5) fruits; and (6) sweets, desserts and soft drinks. Serving size was defined either in “natural” units (e.g., 1 cup of milk, 1 coffee spoon of sugar, 1 egg, 1 apple, etc.) or as an Italian average serving (e.g., 80 g of pasta, 100 g of mixed salad, 175 g of potatoes, 150 g of beef, etc.). Each serving size was converted into the corresponding approximated weight or capacity (Supplementary Table S1). Seasonal variation in fruit and vegetable consumption was also considered to account for the fluctuations within the year. Participants were asked to report the weekly consumption and the duration within the year (in months) for seasonal fruit and vegetables; therefore, the weekly consumption were accordingly reproportioned.

Nutrient content of each food (or recipe) and beverages included in the FFQ were computed using an Italian food composition database (26). This database collected energy and nutrients content for 1,037 foods and beverages. The nutrient content was reported per 100 g of edible matter (or 100 mL for beverages) and thus, the FFQ items were weighted to consider the different weight/capacity of the serving size. The nutritional composition of each recipe (e.g., pasta with tomato sauce) was computed using nutrient content of single ingredients. The Italian food composition database was also used to estimate the total energy intake (kcal/day) for each study subject according to his/her usual diet assessed by FFQ.

### 2.2. Nutritional Functional Diversity indicator (NFD)

We adapted the DeClerk and colleagues’ approach (21) to compute NFD for quantifying diversity in the human diet. The NFD is rooted in the cluster analysis, i.e., a multivariate statistical method that gathers objects according to similarity defined through a function on a set of measured variables (27). There are four steps to calculate NFD: (1) creating a food-nutrient matrix; (2) calculating the food-food distance matrix from the food-nutrient matrix; (3) performing a clustering algorithm on the food-food distance matrix to produce a dendrogram; and (4) calculating individual NFD using the dendrogram.

#### 2.2.1. Step 1: creating a food-nutrient matrix

In the food-nutrient matrix, each row represents a food (i.e., the objects of the cluster analysis) and each column represents a nutrient (i.e., the measured variables of the cluster analysis), such that each cell of the matrix expresses a specific nutrient content of a specific food (as in a food composition table). Our food-nutrient matrix included 70 foods (or recipes) and beverages (all items of the FFQ used to assess diet of subjects) as reported in Supplementary Table S1, and 28 selected nutrients as follows: animal and vegetable proteins, carbohydrates (i.e., water soluble carbohydrates and starch), total fibers, fats (i.e., saturated and monounsaturated fatty acids, linoleic acid, linolenic acid, and other polyunsaturated fatty acids), cholesterol, vitamins (i.e., retinol, thiamine, riboflavin, niacin, vitamin B6, total folate, vitamin C, vitamin D, and vitamin E), minerals (i.e., calcium, iron, potassium, phosphorus, sodium, and zinc), beta carotene equivalent, and lycopene. To account for different scales, nutrients were standardized to have mean of 0 and standard deviation (SD) of 1.

#### 2.2.2. Step 2: calculating the food-food distance matrix from the food-nutrient matrix

The food-food distance matrix contains the pair-wise Euclidean distances between foods, based on their nutrient contents ( K=28 nutrients):


$$d_{ij}=\sqrt{{\left(i_{1}-j_{1}\right)}^{2}+{\left(i_{2}-j_{2}\right)}^{2}+\cdots +{\left(i_{K}-j_{K}\right)}^{2}}\;\mathrm{with}\;k=1,2,\ldots ,K$$

where $d_{ij}$ is the Euclidean distance between foods i and j ; $i_{1}$ is the standardized content of the first nutrient in food i and $j_{1}$ is the standardized content of the first nutrient in food j ; $i_{K}$ and $j_{K}$ are the standardized contents of the last nutrient in foods i and j respectively.

#### 2.2.3. Step 3: performing a clustering algorithm on the food-food distance matrix to produce a dendrogram

Hierarchical clustering of the distance matrix identifies groups (or clusters) of foods according to nutritional similarity. Clusters are created by an unweighted pair group method with arithmetic mean (UPGMA) that uses the following linking algorithm: (1) identifying the minimum distance between any two foods; (2) combining the two foods previously identified as a single pair; (3) re-calculating the Euclidean distance matrix for this new pair and all other foods; (4) identifying the closest pair in the new distance matrix; (5) and so on, until the last two clusters are joint. Clustering results are used to create a dendrogram, a branching diagram that depicts the hierarchical relationship between clusters of foods. Distances between clusters are depicted by branch lengths: longer branches represent greater nutritional dissimilarities between foods or cluster of foods.

#### 2.2.4. Step 4: calculating individual NFD using the dendrogram

**Figure 1:**
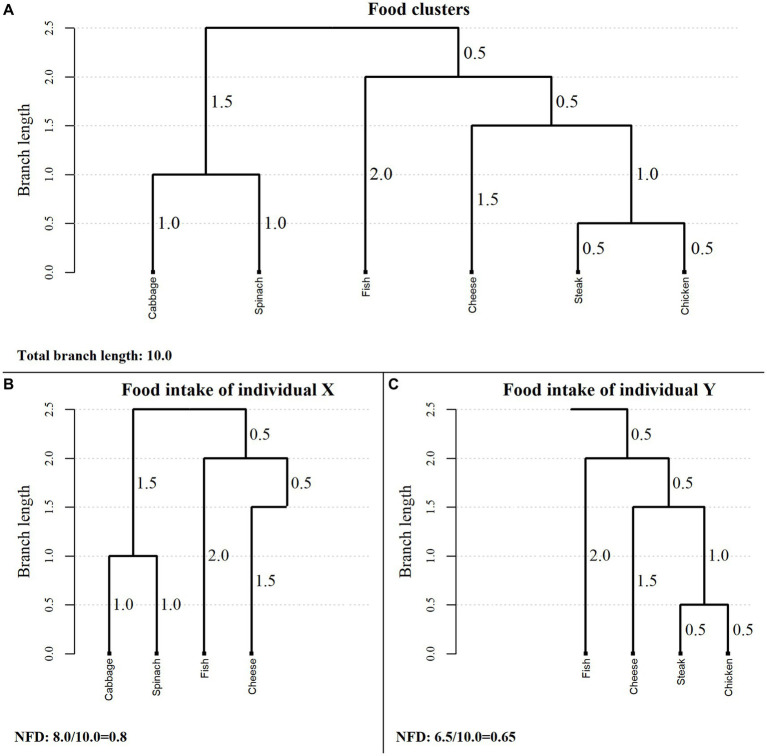
Example of a cluster dendrogram and the corresponding values of the Nutritional Functional Diversity indicator for two hypothetical individuals. NFD, Nutritional Functional Diversity indicator.

The individual NFD is calculated by summing the branch lengths corresponding to individual’s food consumption and dividing by the total branch length of the dendrogram. Thus, NFD is a continuous index defined on the interval (0,1] . To illustrate the computation of NFD from the dendrogram, we propose an example from Luckett et al.’s work (28) adapting it to human diet diversity. Consider the following six foods: cabbage, spinach, fish, cheese, steak, and chicken. Foods were clustered according to the dendrogram reported in Figure 1A. The vertical lines represent the branch length (i.e., similarities between foods or food clusters). For example, steak and chicken are more similar in their nutritional composition than cabbage and spinach, as shown by shorter branch lengths. The denominator of NFD is given by summing all branch lengths of the dendrogram (i.e., total branch length); it corresponds to 10 in our example (Figure 1A). Now, consider the habitual diet of two hypothetical individuals named X and Y. Suppose that X consumes cabbage, spinach, fish, and cheese, whereas Y consumes fish, cheese, steak, and chicken. The NFD for X is given by the sum of branch lengths according to his/her food intake over the total branch length, i.e., 8.0/10.0 = 0.8, 80.0% (Figure 1B). Likewise, NFD for Y is 6.5/10.0 = 0.65, 65.0% (Figure 1C). Thus, the diet of X is more diversified than the diet of Y. Furthermore, note that diets of X and Y would be equivalent according to FVS (i.e., a score of 4 for both individuals).

### 2.3. Assessment of diet quality measures

We used two measures to assess diet quality of study subjects. The first measure was the Mediterranean Diet Score (MDS), developed by Trichopoulou and colleagues (18, 29), which assesses the adherence of individual’s diet to the Mediterranean one. Briefly, the MDS includes 9 dietary components: (1) vegetables; (2) fruit; (3) cereals (including bread and potatoes); (4) legumes; (5) monounsaturated to saturated fatty acids ratio (MUFA/SFA), as a proxy of the consumption of olive oil (30); (6) fish; (7) dairy products (including milk); (8) meat (including poultry, red and processed meat) and; (9) alcohol. A value of 0 or 1 is assigned to each of 9 components using fixed or median sex-specific intakes as cut-offs according to the component considered. In particular, for components more frequently consumed in the Mediterranean diet (i.e., vegetables, fruit, cereals, legumes, MUFA/SFA, and fish), a score of 1 is given if the individual intake is greater or equal to the sex-specific median intake, and 0 otherwise; for components less frequently consumed (i.e., dairy products and meat), a score of 1 is given if the individual intake is lower than the sex-specific median intake, and 0 otherwise. For the alcohol component, a score of 1 is given for an individual consumption ranging from 5 to 25 and 10 to 50 g of ethanol/day for women and men, respectively, and 0 if the individual consumption is outside of these ranges. The MDS is obtained adding up the 9 components’ score, and therefore, it ranges from 0 (no adherence) to 9 (complete adherence) points.

The second measure of diet quality was the Healthy Eating Index (HEI-2015) (31), which assesses the compliance of individual’s diet to the 2015–2020 Dietary Guidelines for Americans (DGA) (32). Briefly, the HEI-2015 includes 13 dietary components: (1) total fruits, (2) whole fruits, (3) total vegetables, (4) greens and beans, (5) whole grains, (6) dairy, (7) total protein foods, (8) seafood and plants proteins, (9) fatty acids, (10) refined grains, (11) sodium, (12) added sugars and, (13) saturated fats. The first 9 components (named adequacy components) represent foods and nutrients that are encouraged to be consumed by the DGA; the remaining 4 components (named moderation components) represent foods and nutrients that are recommended to be limited in the consumption by the DGA. A value from 0 (no compliance) to 10 (complete compliance) is assigned to each of the 13 components using predefined values (named minimum and maximum standards) as cut-offs. Three components consist of two subcategories (i.e., total fruits and whole fruits; total vegetables and greens and beans; and total protein foods and seafood and plant proteins) and each subcategory is assigned a value from 0 to 5. For each component, a proportional score between 0 and 10 (or to 5 for the subcategories) is assigned based on the individual intake ranking in comparison with the standards. As some standards are expressed in cups, we converted the corresponding dietary information from the FFQ (expressed in grams), as reported in our previous application (33). The total score is obtained summing up proportional scores of all components, and therefore, the HEI-2015 is a continuous index ranging from 0 to 100 with higher scores indicating greater compliance to the 2015–2020 DGA.

### 2.4. Statistical analyses

The FVS was computed summing up the number of foods (or recipes) and beverages consumed over a week. For both NFD and FVS, no consumption was defined when a food (or recipe) and beverages has been consumed less than once per week. We used unweighted and weighted Cohen’s kappa statistics (34, 35) to evaluate agreement in study subjects’ classification within quartiles of NFD and FVS. To reflect the degree of disagreement, the weighted kappa uses higher weights for large differences between two categorical ordered variables than weights used for small differences. We used linear weights which are proportional to the discrepancy between subjects’ classification within quartiles of the two diversity indices. The unweighted kappa treats all disagreement equally. Associations between categories of diversity indices (i.e., NFD and FVS) and categories of diet quality measures (i.e., MDS and HEI-2015) were evaluated by unadjusted and adjusted odds ratios (ORs) and corresponding 95% confidence intervals (CIs), estimated by means of multinomial logistic regression models (36). Adjusted models included potentials confounders, namely sex, age (<55; 55–64; ≥65 years), geographical area (North, Center/South), education (<7, 7–11, ≥12 years), year of enrolment in the study (<1994, 1995–1999, ≥2000), BMI (<25.0, 25.0– < 30.0, ≥30.0 kg/m^2^), physical activity (very low, low, high, very high), smoking habit (never, former, current <15, ≥15 cigarettes/day), and energy intake (<1842, 1842–2,249, 2,250–2,737, ≥2,738 kcal/day). The distributions of adjustment factors as well as of MDS and HEI-2015 were reported in Table 1. We fitted separate models for each diversity index (covariates) and each diet quality measure (outcomes).

**Table 1:** Distribution of 7,948 study subjects according to sociodemographic characteristics, year of enrolment, body mass index, physical activity, smoking habit, energy intake, and the adherence to the Mediterranean diet and to the Healthy Eating Index-2015.

Variable	Study subjects	
	*n*	(%)
Sex		
Men	3,660	(46.1)
Women	4,288	(53.9)
Age (years)		
<55	2,974	(37.4)
55–64	2,576	(32.4)
≥65	2,398	(30.2)
Geographical area		
North	6,513	(81.9)
Center/South	1,435	(18.1)
Education (years)^a^		
<7	3,906	(49.3)
7–11	2,052	(25.9)
≥12	1,542	(19.5)
Year of enrolment^a^		
<1994	2,886	(36.3)
1995–1999	3,211	(40.4)
≥2000	1,849	(23.3)
BMI (kg/m^2^)^a^		
<25.0	3,587	(45.1)
25.0– < 30.0	3,259	(41.0)
≥30.0	1,074	(13.5)
Physical activity^a,b^		
Very low	2,427	(31.2)
Low	2,899	(37.3)
High	1,507	(19.4)
Very high	939	(12.1)
Smoking habit^a^		
Never	3,890	(49.0)
Former	1,963	(24.7)
Current <15 cigarettes/day	946	(11.9)
Current ≥15 cigarettes/day	1,133	(14.3)
Energy intake (kcal/day)		
<1,842	1,987	(25.0)
1,842– < 2,250	1,987	(25.0)
2,250– < 2,738	1,987	(25.0)
≥2,738	1,987	(25.0)
MDS (points)		
0–2	915	(11.5)
3–6	6,162	(77.5)
7–9	871	(11.0)
HEI-2015 (values)		
<63.5	2,647	(33.3)
63.5– < 69.1	2,654	(33.4)
≥69.1	2,647	(33.3)

Italy, 1991–2008. ^a^The sum did not add up to the total because of missing values; ^b^Combining levels of physical activity at work/home and during the leisure time.

BMI, body mass index; HEI-2015, Healthy Eating Index-2015; MDS, Mediterranean diet score.

All analyses were conducted using SAS software, version 9.4 (SAS Institute, Inc., Cary, North Carolina, USA).

## 3. Results

We excluded individuals in the top and bottom 2.5% of the estimated sex-specific energy intake distributions (252 men and 214 women) to reduce the effect of implausible extreme values. After these exclusions, the present analysis comprised 3,660 men (46.1%) and 4,288 women (53.9%) as reported in Table 1.

**Figure 2:**
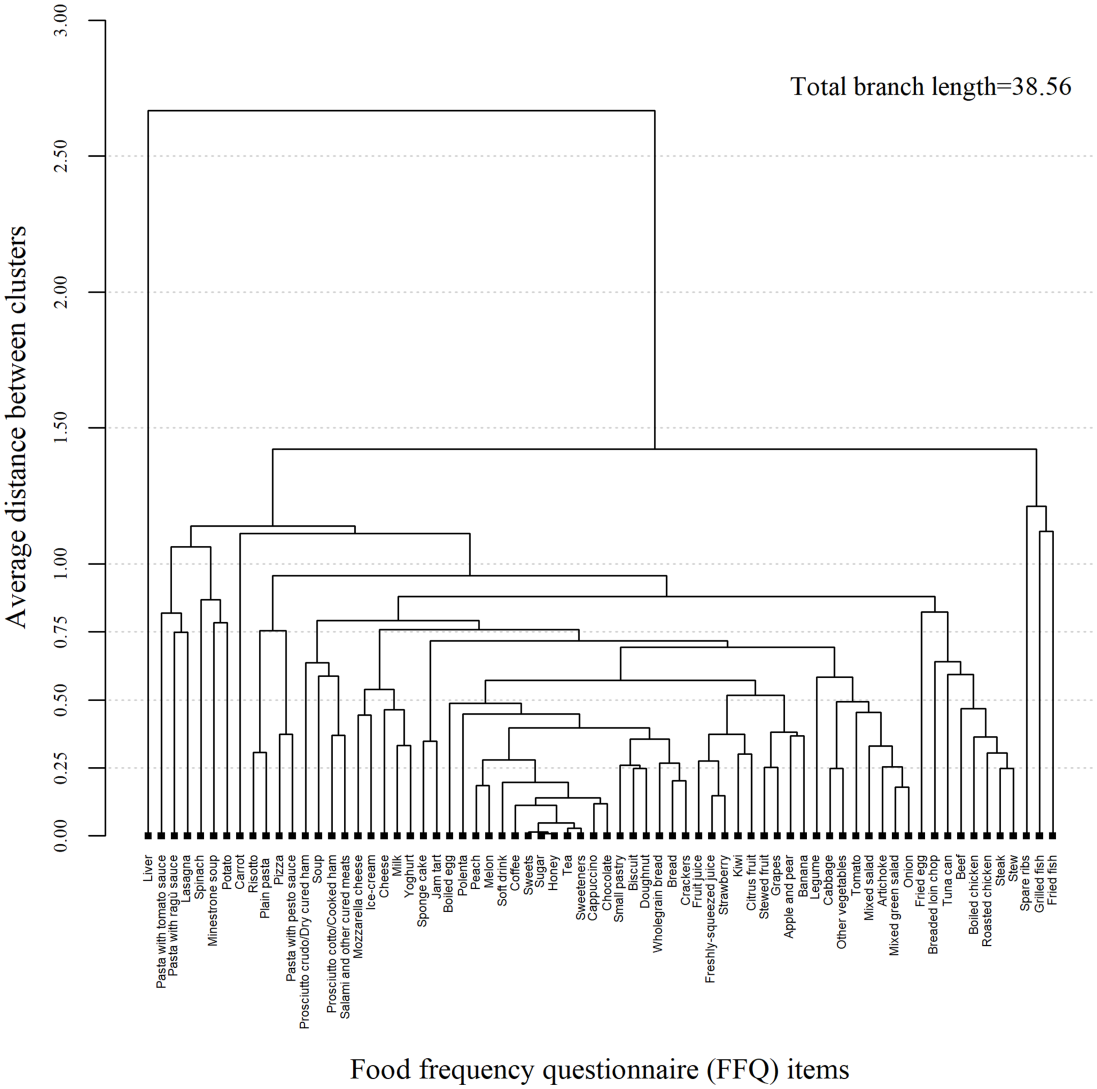
Cluster dendrogram of 70 foods (or recipes) and beverages from an Italian food frequency questionnaire.

The dendrogram reported clusters of foods (or recipes) accounting for a total branch length of 38.56 (Figure 2). In particular, foods with high sugar content (i.e., sweets, sugar, honey, etc.) showed the shortest branch length and were firstly grouped together as reported in the center of the dendrogram. Moving toward the right side of the dendrogram, there were foods rich in starchy carbohydrates. Fruits having high carbohydrate content (i.e., glucose and fructose) were next to the starchy carbohydrate foods and then followed the vegetable cluster. The cluster of foods rich in animal protein located on the right side. To the left side of the dendogram, next to the foods with high sugar content was the cluster of high refined carbohydrates and then next was the dairy food cluster. To the far sides of the dendrogram (both left and right) were residual groups of heterogeneous foods which were grouped to others at the end of clustering process.

**Figure 3:**
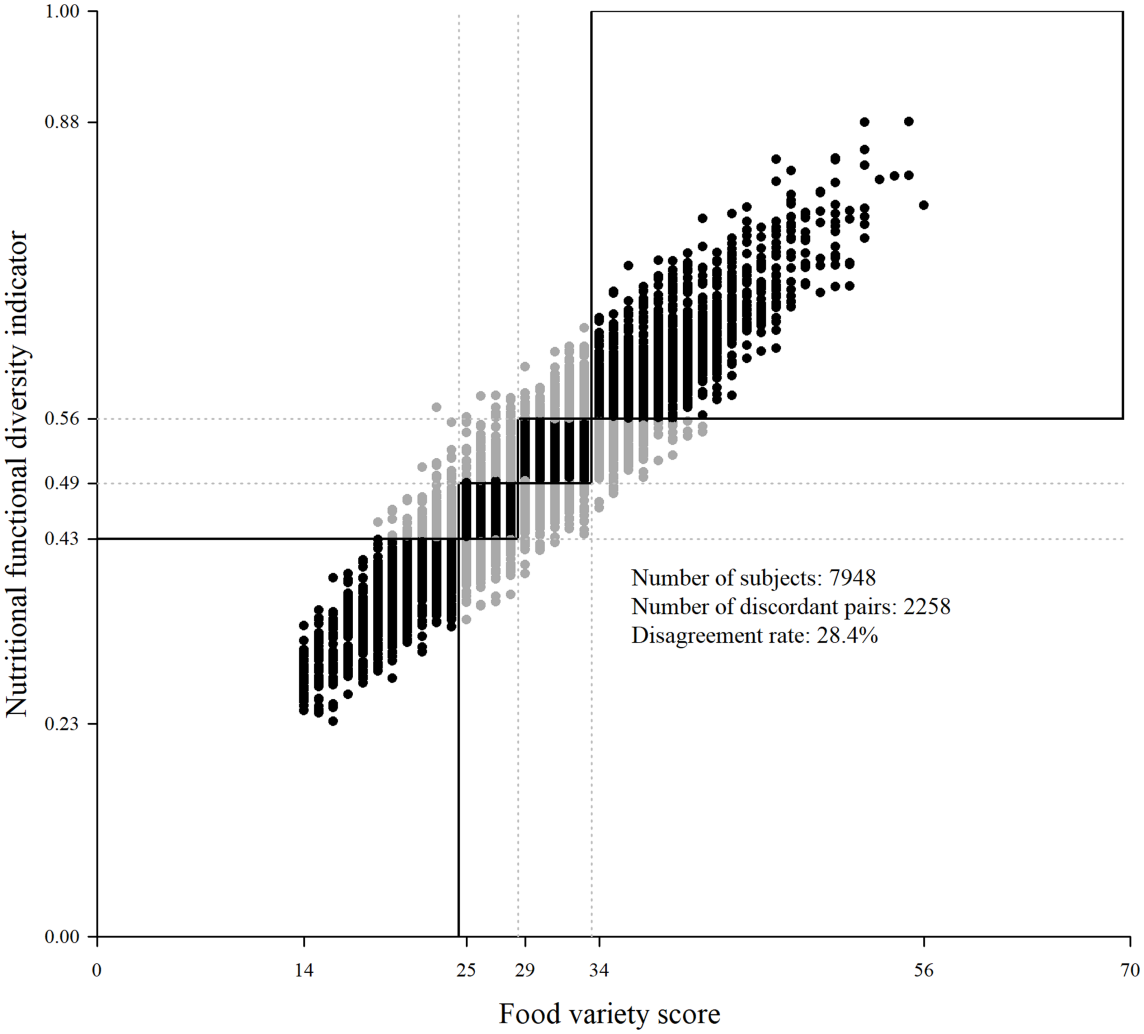
Scatter plot of study subjects according to diversity indices. Italy, 1991–2008. Dotted lines represent quartiles for NFD (*y*-axis) and FVS (*x*-axis); the black boxes represent regions with classification agreement according to quartiles of the two indices; the black circles represent subjects’ classification agreement and gray circles represent subjects’ classification disagreement according to quartiles of the two indices. NFD, Nutritional Functional Diversity indicator; FVS, Food Variety Score.

The NFD ranged from 0.23 to 0.88 with a mean of 0.50 (SD = 0.09), whereas FVS ranged from 14 to 56 with a mean of 29.3 (SD = 6.7). According to quartiles of NFD and FVS, 2285 (28.4%) study subjects were classified differently by the two diversity indices (unweighted Cohen’s kappa = 0.62 and weighted Cohen’s kappa = 0.77; Table 2 and Figure 3). In particular, 4.3% of individuals were classified in the 1st quartile of FVS but in a higher quartile of NFD (i.e., 4.1% were classified in the 2nd quartile and the remaining individuals in a higher one); 4.3% of individuals were in the 2nd quartile of FVS and in 1st quartile of NFD, whereas 4.2% of individuals were in the 2nd quartile of FVS and in a higher quartile of NFD; 6.6% were in the 3rd quartile of FVS and in a lower quartile of NFD, whereas 4.2% were in the 3rd quartile of FVS and in the 4th quartile of NFD; and 4.9% were in 4th quartile of FVS but in a lower quartile according to the NFD.

**Table 2:** Distribution of study subjects according to quartiles of diversity indices.

NFD	FVS				Unweighted Cohen’s kappa	Weighted Cohen’s kappa
	Q1 (<25)	Q2 (25–28)	Q3 (29–33)	Q4 (≥34)		
	*n* (%)	*n* (%)	*n* (%)	*n* (%)		
Q1 (<0.43)	1,629 (20.5)	344 (4.3)	14 (0.2)	0 (0.0)		
Q2 (0.43– < 0.49)	323 (4.1)	1,150 (14.5)	506 (6.4)	8 (0.1)		
Q3 (0.49– < 0.56)	14 (0.2)	314 (4.0)	1,275 (16.0)	384 (4.8)		
Q4 (≥0.56)	1 (<0.1)	16 (0.2)	334 (4.2)	1,636 (20.6)	0.62	0.77

Italy, 1991–2008. df, degree of freedom; FVS, Food Variety Score; NFD, Nutritional Functional Diversity indicator.

Table 3 reported associations between quartiles of the two diversity indices and categories of MDS (i.e., 0–2, 3–6, and 7–9 points). For both NFD and FVS, increasing diversity strongly increased the “risk” for adhering to the Mediterranean diet. Furthermore, unadjusted and adjusted analyses showed a stronger association between NFD and the adherence to the Mediterranean diet. In particular, individuals who were classified in the 4th quartile of NFD showed an adjusted OR of 11.26 (95% CI: 7.88–16.09) which is approximately double than the corresponding OR of 6.80 (95% CI: 4.84–9.54) observed for FVS. A similar pattern was observed for the unadjusted analysis.

**Table 3:** Odds ratios and 95% confidence intervals of the association between diversity indices and the Mediterranean Diet Score.

Diversity index	MDS						
	0–2 points^a^	3–6 points			7–9 points		
	*n* (%)	*n* (%)	OR (95% CI)^b^	OR (95% CI)^c^	*n* (%)	OR (95% CI)^b^	OR (95% CI)^c^
NFD							
Q1	361 (39.3)	1,527 (24.8)	Ref.	Ref.	99 (11.3)	Ref.	Ref.
Q2	263 (28.6)	1,520 (24.7)	1.37 (1.16–1.63)	1.36 (1.14–1.62)	204 (23.2)	2.68 (2.01–3.58)	2.58 (1.92–3.46)
Q3	201 (21.9)	1,537 (25.0)	1.87 (1.55–2.25)	1.80 (1.47–2.19)	249 (28.3)	4.60 (3.44–6.15)	3.89 (2.87–5.27)
Q4	93 (10.1)	1,567 (25.5)	4.69 (3.65–6.03)	4.25 (3.24–4.57)	327 (37.2)	15.15 (10.90–21.05)	11.26 (7.88–16.09)
FVS							
Q1	342 (37.3)	1,508 (24.5)	Ref.	Ref.	117 (13.3)	Ref.	Ref.
Q2	241 (26.3)	1,404 (22.8)	1.29 (1.08–1.54)	1.29 (1.08–1.55)	179 (20.4)	2.10 (1.58–2.79)	2.01 (1.50–2.69)
Q3	209 (22.8)	1,648 (26.8)	1.92 (1.59–2.31)	1.84 (1.51–2.24)	272 (30.9)	3.89 (2.94–5.15)	3.26 (2.42–4.38)
Q4	126 (13.7)	1,591 (25.9)	3.46 (2.76–4.34)	3.16 (2.45–4.08)	311 (35.4)	9.13 (6.74–12.36)	6.80 (4.84–9.54)

Italy, 1991–2008. ^a^Reference category; ^b^Unadjusted estimates; ^c^Adjusted estimates for sex, age (<55; 55–64; ≥65 years), geographical area (North; Center/South), education (<7; 7–11; ≥12 years), year of enrolment (<1994; 1994–1999; ≥2000), BMI (<25; 25– < 30; ≥30 kg/m^2^), physical activity (very low; low; high; very high), smoking habit (never, former, current <15; current ≥15 cigarettes/day); and energy intake (<1,842; 1,842– < 2,240; 2,250– < 2,738; ≥2,738 kcal/day).

BMI, body mass index; CI, confidence interval; FVS, Food Variety Score; MDS, Mediterranean Diet Score; NFD, Nutritional Functional Diversity indicator; OR, odds ratio.

Table 4 reported associations between quartiles of the two diversity indices and categories of HEI-2015 (i.e., <63.5, 65.5- < 69.1, ≥69.1). Although less marked, there was an increasing association between quartiles of both diversity indices and the adherence to the HEI-2015. Individuals in the 4th quartile of NFD showed a risk of 2.86 (95% CI: 2.39–3.42) to adhere to the higher category of HEI-2015 which is slightly higher than the corresponding risk observed for FVS (OR = 2.72; 95% CI: 2.27–3.26).

**Table 4:** Odds ratios and 95% confidence intervals of the association between diversity indices and the Healthy Eating Index-2015.

Diversity index	HEI-2015						
	<63.5^a^	63.5– < 69.1			≥69.1		
	*n* (%)	*n* (%)	OR (95% CI)^b^	OR (95% CI)^c^	*n* (%)	OR (95% CI)^b^	OR (95% CI)^c^
NFD							
Q1	870 (32.9)	550 (20.7)	Ref.	Ref.	567 (21.4)	Ref.	Ref.
Q2	678 (25.6)	638 (24.0)	1.49 (1.28–1.73)	1.58 (1.35–1.82)	671 (25.3)	1.52 (1.31–1.76)	1.65 (1.41–1.92)
Q3	584 (22.1)	676 (25.5)	1.83 (1.57–2.14)	2.06 (1.75–2.43)	727 (27.5)	1.91 (1.64–2.22)	2.28 (1.93–2.68)
Q4	551 (19.5)	790 (29.8)	2.43 (2.08–2.83)	3.06 (2.57–3.65)	682 (25.8)	2.03 (1.74–2.37)	2.86 (2.39–3.42)
FVS							
Q1	845 (31.9)	567 (21.4)	Ref.	Ref.	555 (21.0)	Ref.	Ref.
Q2	652 (24.6)	556 (20.9)	1.27 (1.09–1.48)	1.35 (1.15–1.58)	616 (23.3)	1.44 (1.23–1.68)	1.56 (1.33–1.83)
Q3	586 (22.1)	753 (28.4)	1.92 (1.65–2.23)	2.19 (1.86–2.57)	790 (29.8)	2.05 (1.76–2.39)	2.51 (2.13–2.96)
Q4	564 (21.3)	778 (29.3)	2.06 (1.77–2.39)	2.67 (2.24–3.19)	686 (25.9)	1.85 (1.59–2.16)	2.72 (2.27–3.26)

Italy, 1991–2008. ^a^Reference category; ^b^Unadjusted estimates; ^c^Adjusted estimates for sex, age (<55; 55–64; ≥65 years), geographical area (North; Center/South), education (<7; 7–11; ≥12 years), year of enrolment (<1994; 1994–1999; ≥2000), BMI (<25; 25– < 30; ≥30 kg/m^2^), physical activity (very low; low; high; very high), smoking habit (never, former, current <15; current ≥15 cigarettes/day); and energy intake (<1,842; 1,842– < 2,240; 2,250– < 2,738; ≥2,738 kcal/day).

BMI, body mass index; CI, confidence interval; FVS, Food Variety Score; MDS, Mediterranean Diet Score; NFD, Nutritional Functional Diversity indicator; OR, odds ratio.

## 4. Discussion

We adapted the NFD to quantify diversity in the human diet. We firstly evaluated study subjects’ distributions across quartiles of NFD and FVS showing that more than one out of four subjects (i.e., 28.4%) were differently classified within quartiles of the two diversity indices. To assess which diversity index reflected a higher diet quality, we estimated their associations with MDS and HEI-2015. We observed stronger associations between NFD and both MDS and HEI-2015.

The NFD introduced nutritional (dis)similarity of foods consumed in calculating diversity. This led to a different diversity level of more than 25% of our sample using NFD instead of FVS. The NFD tries to answer the question: “What is the nutritional dissimilarity of the number of different foods consumed?”, whereas FVS and other diversity indices based on counting approach answer the question: “What is the number of different foods (or food groups) consumed?”. In high-income country populations that are characterized by complex dietary patterns, the simply counting approach could lead to a poor evaluation of diversity and in turn of overall diet quality. Over the time, several composite measures of diet quality have been proposed for these populations (13, 19). Generally, these measures were based on a multidimensional concept of diet quality including: diversity (both across and within food or food groups), adequacy (sufficiency of dietary components consumption compared to recommended energy requirements), moderation (restriction of specific nutrient or food intake to prevent harmful effect on health), and overall balance (the proportionality of macronutrient intake) (12). The stronger associations observed between NFD and both MDS and HEI-2015 may reflect a higher ability of NFD than FVS to assess diet quality, measuring one of its key components: diet diversity. A recent systematic scoping review aimed to provide the link of existing diversity indices and their association to health outcomes in adolescents and adults, concluding that the ability of available diversity indices to reflect diet quality is principally limited. The authors emphasized the inappropriate use of available indices to assess overall diet quality also in low- and middle-income countries (37). Alkerwi previously reviewed the concept of diet quality and concluded that an integrated approach that combines all different dimensions of diet quality is needed to successfully measure it. In addition, the author raised the importance of considering both nutritional characteristics and other facets of diet quality such as food safety, organoleptic properties, and cultural aspects when designing diet quality measures (12).

Although FVS is easy to compute (simply adding up the foods consumed), it fails to discriminate between diets with the same number of foods consumed but with different nutritional compositions. Incorporating the nutritional (dis)similarity of food consumed, NFD reflects a higher diet quality, providing a different diversity level than FVS. Nonetheless, it requires a more complex calculation (i.e., a cluster analysis). In addition, NFD depends on the choice of nutrients included in the cluster analysis. Different nutrients will produce different clusters of foods, as will different values of NFD. We proposed a comprehensive list of essential nutrients which could also be applied in populations with different taste preferences and food availability than the Italian one. However, we do not exclude the possibility of using different sets of nutrients in other specific contexts. Moreover, the choice of foods to include in the cluster analysis may also impact on the calculation of NFD limiting the generalizability of this index. As FVS, the NFD does not consider frequencies of consumption which in turn limits the overall evaluation of diet quality. This could be supported by weaker associations for both diversity indices and HEI-2015 which is designed on density-based amounts. In addition, dietary behaviors may change during time and the long enrolment period of study subjects included in this analysis could have impacted in the assessment of diversity. However, changes in diet may affect to a greater extent the frequency of consumption than the selection of foods themselves. Since frequency of consumption is not accounted for in NFD computation, we expect a limited impact of dietary changing through time on the present results. We evaluated the association between NFD and FVS with two recognized measures of diet quality, it could be interesting to assess the association with other existing diet quality measures. In addition, simulation studies of typical diets could be carried out to assess in a more comprehensive and generalizable way the associations between NFD and other diet quality measures. Furthermore, it would be useful to apply our proposal in other study populations to confirm results of the present study and to validate our methodology in different contexts. Lastly, NDF may have limited application for populations in low-income countries due to lack of individual level of food consumption data, as well the necessary dietary reference data (e.g., food composition tables). For these populations the use of simple indices could constitute valid and suitable options. However, low- and middle-income countries are increasingly collecting individual level dietary data and therefore application of the NFD method to assess diversity may be more feasible in the future. In addition, NFD could better discriminate subjects’ group of high-income countries (who generally consumed a wider range of foods with different nutritional composition) and investigate the relationship between each identified group and the development of disease.

## 5. Conclusion

Our findings indicate that NFD provided different diversity levels of FVS for more than a quarter of our sample. In addition, NFD was more strongly associated with a higher adherence to the Mediterranean diet and Healthy Eating Index-2015 providing evidence of a greater ability of this tool to assess diet quality evaluating diversity.

## Data availability statement

The data analyzed in this study is subject to the following licenses/restrictions: participating hospitals are owners of data. Anonymized patient data are available for use in collaborative studies to researchers upon reasonable request to the corresponding author. Data will be provided following the review and approval of a research proposal (including a statistical analysis plan) and the completion of a data‑sharing agreement. Requests to access these datasets should be directed to matteo.dimaso@unimi.it.

## Ethics statement

The studies involving humans were approved by Hospitals of participating centres. The studies were conducted in accordance with the local legislation and institutional requirements. The participants provided their written informed consent to participate in this study.

## Author contributions

MF conceptualized the methodological idea. MDM and FB further developed the methodological idea, performed the analyses, and drafted the manuscript. JP helped in the statistical methodological approach. LP helped with the nutritional issues and interpretations. CLV managed data collection and helped to review the final version of the manuscript. LDM helped to interpret the results. All authors critically reviewed and approved the final version of the manuscript.
