# Supplementary material for: A novel approach to assess diet diversity: a development of the Nutritional Functional Diversity indicator
Source: Front Nutr. 2023 Oct 19;10:1170831. doi: 10.3389/fnut.2023.1170831 (PMC10620300; doi:10.3389/fnut.2023.1170831)
Supplement: Supplementary file 1 [file Table_1.DOCX]

Supplementary Material

A novel approach to assess diet diversity: a development of the Nutritional Functional Diversity indicator

Matteo Di Maso^*^, Francesca Bravi, Jerry Polesel, Linia Patel, Carlo La Vecchia, Monica Ferraroni

*** Correspondence:** Matteo Di Maso, [matteo.dimaso@unimi.it](mailto:matteo.dimaso@unimi.it)

**Supplementary table 1.** Description, serving size, and approximately weight/capacity of foods (or recipes) and beverages from an Italian food frequency questionnaire.

| **Food frequency questionnaire item** | **Serving size** | **Approximately**  **weight/capacity** |
| --- | --- | --- |
|  |  |  |
| Milk (*whole, skimmed or partially skimmed milk*) | 1 cup | 225 ml |
| Yoghurt | 1 cup | 125 ml |
| Cappuccino | 1 cup | 125 ml |
| Coffee (*espresso or moka coffee, including decaffeinated*) | 1 cup | 50 ml |
| Tea | 1 cup | 125 ml |
| Sugar | 1 coffee spoon | 3 g |
| Sweeteners (*saccharin and other sweeteners*) | 1 sachet or tablet | 1.25 g |
| Bread | 1 portion or 1 slice | 50 g |
| Wholegrain bread | 1 portion or 1 slice | 50 g |
| Crackers (*crackers, bread sticks or crisp bread*) | crackers: 1 pack  bread sticks: 5 sticks  crisp bread: 3 slices | 30 g |
| Polenta (*cooked polenta*) | 1 portion or 1 slice | 100 g |
| Pizza (*pizza margherita*) | 1 pizza | 200 g |
| Risotto (*risotto with vegetable soup*) | 1 portion | 80 g |
| Plain pasta (*boiled pasta*) | 1 portion | 80 g |
| Pasta with tomato sauce | 1 portion | 80 g |
| Pasta with ragù sauce | 1 portion | 80 g |
| Pasta with pesto sauce | 1 portion | 80 g |
| Lasagna (*lasagna, cannelloni or tortellini with meat*) | 1 portion | 250 g |
| Soup (*soup with pasta and vegetables*) | 1 portion | 250 g |
| Minestrone soup (*minestrone soup or pasta with beans*) | 1 portion | 250 g |
| Boiled egg (*boiled or soft-boiled egg*) | 1 egg | 65 g |
| Fried egg (*fried egg or omelette*) | 1 egg or omelette | 100 g |
| Boiled chicken (*boiled chicken or turkey*) | 1 portion | 200 g |
| Roasted chicken (*roasted chicken, turkey or rabbit*) | 1 portion | 200 g |
| Steak (*steak, fillet steak, and roast-beef*) | 1 portion | 120 g |
| Beef (*boiled beef*) | 1 portion | 150 g |
| Stew (*stew, braised, and meat balls from beef or veal*) | 1 portion | 150 g |
| Breaded loin chop (*breaded loin chop from beef or veal*) | 1 portion | 120 g |
| Spare ribs (*spare ribs pork*) | 1 portion | 150 g |
| Liver (*liver from chicken, turkey, rabbit, cow or pork*) | 1 portion | 150 g |
| Prosciutto crudo/Dry cured ham | 6 slices | 50 g |
| Prosciutto cotto/Cooked ham | 3 slices | 50 g |
| Salami and other cured meats | 5 slices | 50 g |
| Grilled fish | 1 portion | 150 g |
| Fried fish | 1 portion | 150 g |
| Tuna can (*tuna, mackerel, or sardines in olive oil*) | 1 can | 80 g |
| Mozzarella cheese (*mozzarella or ricotta cheese*) | 1 portion | 100 g |
| Cheese (*parmigiano and other aged cheeses*) | 1 portion or 1 slice | 100 g |
| Legume (*peas, beans, chickpeas or lentils cooked*) | 1 portion | 100 g |
| Mixed green salad (*lettuce and radicchio with olive oil*) | 1 portion | 50 g |
| Carrot (*raw carrots with olive oil and cooked carrots with butter or olive oil*) | 1 portion | 100 g |
| Onion | 1 portion | 80 g |
| Potato (*boiled, fried and roasted potatoes*) | 1 portion | 175 g |
| Artichoke (*artichoke cooked with butter or olive oil*) | 1 portion | 125 g |
| Cabbage (*cabbage, cauliflower, broccoli, brussels sprouts or turnip greens cooked with butter or olive oil*) | 1 portion | 125 g |
| Spinach (*cooked spinach with butter or olive oil*) | 1 portion | 200 g |
| Tomato (*raw tomatoes with olive oil*) | 1 portion | 150 g |
| Mixed salad (*mixed salad with lettuce, carrots, cucumbers, and peppers*) | 1 portion | 100 g |
| Other vegetables (*courgettes, aubergines or peppers cooked with butter or olive oil*) | 1 portion | 150 g |
| Apple and pear | 1 fruit | 150 g |
| Banana | 1 fruit | 180 g |
| Kiwi | 1 fruit | 90 g |
| Stewed fruit | 1 portion | 125 g |
| Freshly-squeezed juice | 1 glass | 150 ml |
| Fruit juice | 1 can | 125 ml |
| Honey (*honey or marmalade*) | 1 coffee spoon | 3 g |
| Citrus fruit (*oranges, grapefruit, or tangerine*) | orange: 1 fruit  grapefruit: ½ fruit  tangerine: 3 fruits | 150 g |
| Peach (*peaches, apricot or plum*) | peach: 1 fruit  apricot: 2 fruits  plum: 2 fruits | 100 g |
| Melon | 2 slices | 75 g |
| Grapes | 1 bunch | 230 g |
| Strawberry (*strawberries, cherries, and other red fruit*) | 1 cup | 150 g |
| Biscuit | 7 biscuits | 50 g |
| Doughnut | 1 doughnut | 50 g |
| Small pastry (*small pastry filled with cream*) | 3 pastry | 50 g |
| Sponge cake | 1 slice | 100 g |
| Jam tart | 1 slice | 100 g |
| Chocolate | 1 square | 10 g |
| Soft drink | 1 glass | 150 ml |
| Sweets | 1 portion | 3 g |
| Ice-cream | 1 portion | 100 g |
|  |  |  |
